# Translational repression in the pathogenesis of FUS- and C9orf72-dependent ALS

**DOI:** 10.1186/2193-1801-4-S1-L51

**Published:** 2015-06-12

**Authors:** Mauro Cozzolino, Simona Rossi, Gianluca Cestra

**Affiliations:** Institute of Translational Pharmacology, CNR, Rome, Italy; Institute of Molecular Biology and Pathology, CNR, Rome, Italy

**Keywords:** ALS, RNA trafficking, stress granules

The major focus of ALS research has recently moved to RNA control of motor neuron functions, as most of the newly identified genes, that alone account for more than half of ALS familial cases, are clearly associated to RNA regulation. These include FUS and TDP43, two DNA/RNA binding proteins with a role in the regulation of RNA transcription, splicing, transport and translation, and C9orf72, a gene that is marked by the presence, in carriers, of an highly expanded GGGGCC repeat that is believed to provide the mutant gene of an acquired, toxic feature by an RNA-dependent gain of function mechanism. Thus, RNA dys-metabolism is likely to represent a central issue in ALS pathogenesis. Yet, whether a specific step of RNA processing is particularly affected in ALS motor neurons is unclear. We have recently obtained evidence that a prominent effect of FUS and C9orf72 expression is the induction of stress granules-associated translational repression. In this presentation I will discuss our recent work on the molecular mechanisms underlying these effects and their potential relevance in ALS pathogenesis.

